# Comparison of amyloid and tau pathology in the small and large intestines of individuals with Alzheimer's disease, Parkinson's disease, and Dementia with Lewy bodies

**DOI:** 10.17912/micropub.biology.002056

**Published:** 2026-07-15

**Authors:** Angela M. Floden, Gunjan D. Manocha, Natalia I. Frolov, Andrea E. Lerick, Colin K. Combs

**Affiliations:** 1 Department of Biomedical Sciences, School of Medicine and Health Sciences, University of North Dakota, Grand Forks, ND, United States; 2 Department of Geriatrics, School of Medicine and Health Sciences, University of North Dakota, Grand Forks, ND, United States

## Abstract

Alzheimer's disease (AD) brains are characterized by accumulations of neurofibrillary tangles and amyloid β (Aβ) plaques. Since enteric neurons express tau and the amyloid precursor protein (APP), we asked whether neurofibrillary tangles and Aβ aggregates were present in AD intestines compared to healthy controls and individuals with Parkinson's disease (PD) and Dementia with Lewy Bodies (DLB). Neuron-like APP and Aβ immunoreactivities were observed in all groups with no observable plaques. No tangle-like structures were observed in any group although p-Ser 396/404 tau immunoreactivity was seen. The enteric nervous system appears to be protected from developing tangle and plaque pathology in AD.

**Figure 1. APP, Aβ, and p-tau immunoreactivity in the intestines of age-matched controls, AD, PD, and DLB donors:** (A) Representative images of Aβ immunoreactivity scores of 0, 1, 2, and 3 are shown to provide context for scoring criteria. Arrowheads indicate representative positive staining. Scale bar 500µm. (B) Neuron-like APP immunoreactivity was observed in the submucosal and myenteric plexi in both male and female tissue from all groups. Representative images from the sigmoid colon are shown. Arrowheads indicate positive staining. Scale bar 50µm **.** (C) Neuron-like Aβ immunoreactivity was observed in the subcutaneous and myenteric plexi of all groups throughout the intestines. Representative images from the sigmoid colon are shown. Arrowheads indicate positive staining. Scale bar 50µm **.** There were no significant differences in Aβ staining abundance between groups within the duodenum, jejunum, ileum, or colon in (D) male or (E) female comparisons. Immunoreactivities for every individual across the intestinal regions are color coded. (F) Neuron-like phospho-tau (PHF-1) immunoreactivity was observed in the submucosal and myenteric plexi of all groups throughout the intestine. Representative images from the sigmoid colon are shown. Arrowheads indicate positive staining. Scale bar 50µm **.** (G) There were no significant differences in PHF-1 staining abundance between groups within the duodenum, ileum, jejunum, or colon in either male or female comparisons. Immunoreactivities for every individual across the intestinal regions are color coded. (H) Sparse to no phospho-tau (AT8) immunoreactivity was observed in any intestinal region across the groups in male or female comparisons. Immunoreactivities for every individual across the intestinal regions are color coded. **Table 1. Donor Samples.**

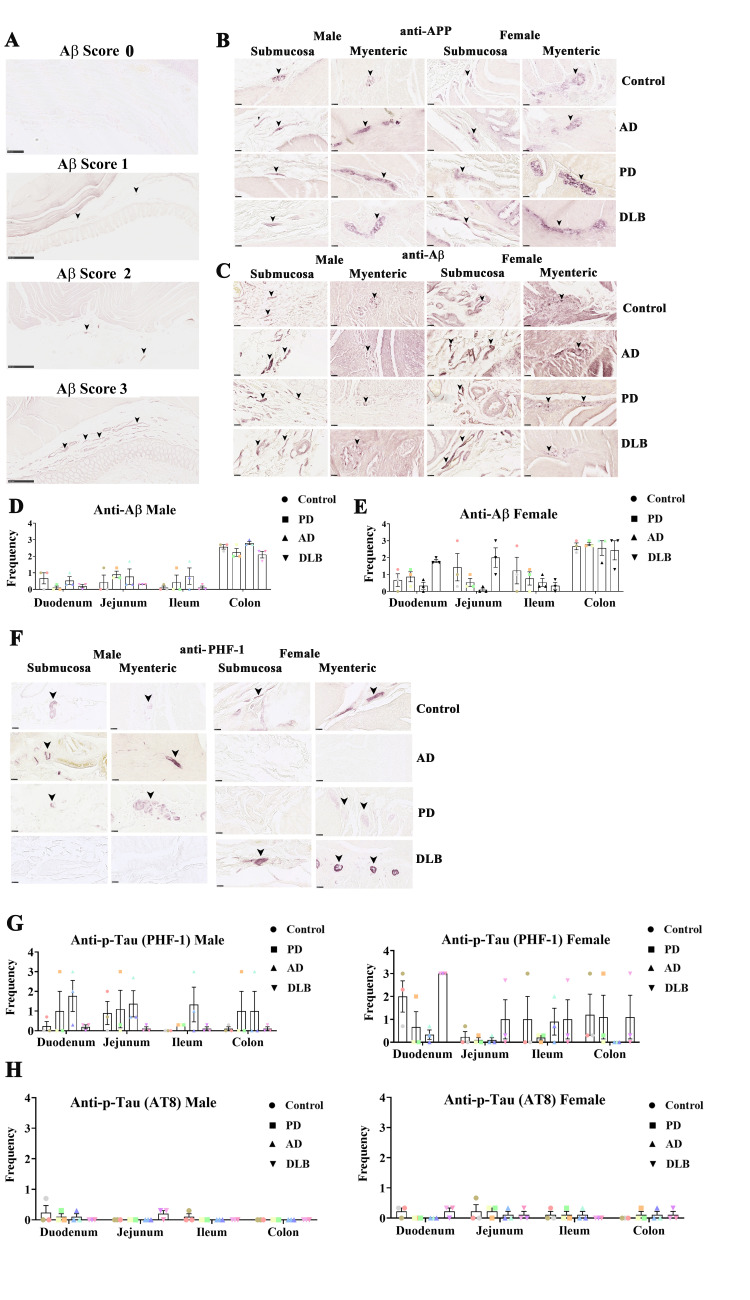



**Table:** 

Clinical Diagnosis	Braak Stage	PMI	Sex	Age
Control	III	2.07	M	80
Control	II	2.92	M	73
Control	III	2.5	M	87
Control	III	3.55	F	87
Control	I	4.12	F	82
Control	III	3.5	F	93
AD	V	2.95	M	82
AD	VI	3	M	80
AD	V	3.83	M	75
AD	VI	2.7	F	89
AD	V	3.08	F	76
AD	VI	3.85	F	81
PD	IV	2.42	M	80
PD	IV	5.42	M	85
PD	II	3.37	M	69
PD	II	3.16	F	79
PD	IV	2.08	F	87
PD	IV	4	F	88
DLB	V	3	M	68
DLB	V	2.8	M	95
DLB	V	2.29	M	80
DLB	VI	2.5	F	68
DLB	VI	2.33	F	81
DLB	VI	3.48	F	62

## Description

Our lab has previously demonstrated the expression of amyloid precursor protein (APP), from which amyloid beta (Aβ) is generated, in the intestines of both transgenic mouse models of Alzheimer's disease (AD) and human AD cases (Puig, KL et al., 2013). Moreover, we and others have also observed intraneuronal as well as epithelial APP and Aβ immunoreactivity in both human and mouse intestines (Puig, KL et al., 2015; Manocha, GD et al., 2019; Jin, J et al., 2023; Li, G et al., 2023; Galloway, S et al., 2007; Galloway, S et al., 2009). In addition, prior studies have demonstrated Aβ extravascular and mucosal staining in rodent models, as well as in human intestines, with more robust staining in AD patients and AD models compared to controls (Manocha, GD et al., 2019; Liu, G et al., 2023; Joachim, CL et al., 1989). This suggests that Aβ deposition may occur in the intestines as a novel peripheral manifestation of disease.

Rodent studies have demonstrated increased anti-phospho-tau immunoreactivity in rat enteric neurons in the ileum and colon during hypothermic stress, as assessed using the AT8 antibody (Chiocchetti, R et al., 2021). Another study examining human appendix tissue also noted myenteric plexus phospho-tau immunoreactivity using the AT8 antibody (Zinnen, AD et al., 2022). Phospho-tau immunoreactivity, using both the AT8 and PHF-1 antibodies, demonstrated phosphatase-resistant phosphorylation in colon enteric neurons of control, Parkinson's disease (PD), and Progressive Supranuclear Palsy samples, with no difference in levels across the groups (Lionnet, A et al., 2018).

The purpose of this study was twofold. We first aimed to determine whether any Aβ plaque or phospho-tau tangle pathology occurred in different regions of the AD intestine. Second, we asked whether amyloid or tangle pathology was unique to AD intestines compared to tissue from control, PD, and DLB individuals.

We first assessed the distribution of APP immunoreactivity in the various regions of the intestine. As expected, APP staining was observed in all groups and in all intestinal areas of the submucosal and myenteric plexi, consistent with a neuronal-like localization based on the morphological presentation of the stained cell bodies and fibers in the submucosa and muscularis mucosa consistent with the location of the submucosal and myenteric plexi ( Fig. 1A ).

Next, we assessed whether plaque-like immunoreactivity was present in any region or group. Although no plaque-like staining pattern was observed in any area of the intestine or any group, there was a consistent punctate Aβ staining pattern observed once again in the submucosal and myenteric plexi with more abundant immunoreactivity in the colon ( Fig. 1B ). There were no significant differences between AD, DLB, or PD tissue when compared to controls in any intestinal region. However, colons, in general, demonstrated more robust staining in all groups.

To assess tangle pathology, the anti-phospho-tau antibody, PHF-1, was used to stain the tissues. There was little evidence of tangle or dystrophic neurite pathology in any group or intestinal region. However, once again, there was a clear demonstration of neuron-like submucosal and myenteric plexus immunoreactivity consistent with neuronal expression based on cell body and fiber morphology ( Fig. 1C ). The degree of phospho-tau immunoreactivity, comparing control to all disease intestines, was not significantly different in any region. An additional phosphorylated tau antibody, AT8, was next used for immunostaining. Notably, there was very little to no AT8 immunoreactivity in the intestines with no significant differences across regions when comparing all groups to control samples ( Fig. 1C ).

Although enteric neurons demonstrated APP immunoreactivity, no obvious plaque-like deposits were observed. However, intraneuron-like Aβ immunoreactivity suggests that the peptide is likely generated in the intestine, although it is not deposited extracellularly. We and others have observed intestinal Aβ immunoreactivity in mouse models and human samples (Puig, KL et al, 2015; Manocha, GD et al, 2019; Joachim, CL et al., 1989). One possibility for the absence of Aβ plaque-like deposition is that the robust multi-immune cell environment of the intestine provides a greater opportunity for Aβ clearance. Intestinal macrophages and macrophages in general have been reported to have the ability to phagocytose Aβ, perhaps more robustly than their brain microglia counterparts (Liu, G. et al., 2023; Zhao, L. et al., 2009). Additionally, it is possible that enteric neurons do not secrete Aβ into the extracellular space. Alternatively, perhaps Aβ is produced and secreted preferentially by epithelial cells as we and others have suggested (Galloway, S. et al., 2007; Galloway, S. et al., 2009; Wu, S. et al., 2022; Puig, KL et al., 2015; James, AP et al., 2003; Pallebage-Gamarallage, MM et al., 2009; Pallebage-Gamarallage, MM et al., 2012). Lack of plaque immunoreactivity may also be related to alternative APP isoform expression in the intestines versus brains or limited secretase expression in the intestines (Jin, J. et al., 2023). These findings suggest that Aβ production, aggregation, and clearance may be regulated differently in the intestines compared to the brain and the peptide may not exhibit AD-selective behavior in the intestines.

We examined neurofibrillary tangle pathology in the AD and control intestines using two different phospho-epitope-specific antibodies, PHF-1 and AT8. Surprisingly, we observed little to no AT8 immunoreactivity in any region of the intestine. The PHF-1 antibody demonstrated immunoreactivity, as expected, in neuron-like cell bodies of both the submucosal and myenteric plexi. However, there were no observable neurofibrillary tangles, as are commonly found in the brain. Nevertheless, future work to determine the proteinase K resistance of the intestinal tau staining would be useful to better compare to brain presentation of the protein. It is challenging to explain why the tau phosphorylation patterns differ with the PHF-1 phospho-Ser 396/404 epitope appearing more abundantly in the intestine (Greenberg, S. et al., 1992; Otvos, L. et al., 1994). It is possible that enteric neurons express unique tau isoforms that are more resistant to maintained phosphorylation and paired helical filament formation (Lionnet, A. et al., 2018). Alternatively, additional tau phosphorylation sites or post-translational modifications may contribute uniquely to tau aggregation in the brain (Parra Bravo, C. et al., 2024). Similar to the plaque comparisons, these data demonstrate that tangle formation in the brains and intestines of AD patients may be unique, and intestinal changes might not be a reliable indicator of disease progression.

Further work with a larger sample size, comparing different tau phospho-epitopes, post-translational modifications, and tau isoforms, as well as APP isoforms and beta and gamma secretases, will provide better support for any differences in APP and tau behavior in neurons from either the enteric or central nervous systems. Determining whether intestinal pathology or dysfunction can be used to differentiate AD from age-matched control or other disease intestines will require additional investigation.

## Methods

**Human Tissue Samples**

Tissues from autopsied donors were provided by Banner Sun Health Research Institute (SHRI), Sun City, Arizona. Human tissue use was approved by the University of North Dakota (UND) IBC2304-0009 Institutional Biosafety Committee (IBC). Paraffin-embedded tissue sections (6µm) from three male and three female samples each of control (ND), Parkinson's Disease (PD), Alzheimer's Disease (AD), and Dementia with Lewy Bodies (DLB) disease-matched mid-temporal gyrus (MTG), duodenum, jejunum, ileum, and sigmoid colon samples were obtained. Donor ages ranged between 62 and 95 years of age, Braak stages I-VI, and PMI from 2.07-4.12 hours (Table 1). The tissue was collected as previously described (Beach, TG et al., 2015; Beach, TG et al., 2010; Beach, TG et al., 2008; Walker, DG et al., 2016).

**Immunohistochemistry**

Paraffin-embedded sections were deparaffinized using Histoclear II (National Diagnostics, Atlanta, GA) and passaged through an increasingly diluted gradient of ethanol/water. We then performed standard immunohistochemistry on all groups, using three male and three female samples per group. To recognize tau changes, an anti-phosphorylated tau antibody (AT8 clone, cat # MN1020) from Life Technologies (Chicago, Illinois) was used at a 1:1000 dilution after an 80% formic acid antigen retrieval step for 20 minutes. An anti-Amyloid β antibody, 4G8, from BioLegend (cat. # 800703) (San Diego, CA), was used at a 1:750 dilution after antigen retrieval with 80% formic acid for 20 minutes, followed by two rinses with water. The Anti-APP antibody, Y188 (cat. # ab32136) from Abcam was used at a 1:500 dilution. The anti-phospho-tau antibody, PHF-1, was a kind gift from the late Dr. Peter Davies of Albert Einstein University, NY. PHF-1 immunohistochemistry (1:500) required antigen retrieval using 80% formic acid for 20 minutes, followed by two rinses with water. Biotinylated secondary antibodies were used in conjunction with the Vector Vectastain Elite ABC kit (PK-6100) to visualize antibody binding in each tissue, followed by the application of Vector VIP (PK-4600) as the chromogen, both from Vector Laboratories (Burlingame, CA). Tissue sections were dehydrated in an ethanol series of increasing purity, finishing with absolute ethanol. Then, two incubations in Histoclear II were followed by mounting with Permount (Fisher Scientific, Pittsburgh, PA) using a Leica CV5030 Coverslipper (Buffalo Grove, IL).

**Semi-quantitative immunohistochemical analysis**

To compare staining across intestinal regions and tissue groups, we employed a semi-quantitative approach to analyze our immunohistochemistry results based upon our prior work as well as that of others (Puig, KL et al., 2015; Beach, TG et al., 2008). Briefly, immunoreactivity for each antibody was quantified by three blinded examiners, who assigned a score of 0 for no staining, 1 for sparse staining, 2 for moderate staining, and 3 for frequent staining. The three blinded scores were averaged from 3 sections for each intestinal region in each group. Stained tissue slides were scanned with a Nanozoomer 2.0HT slide scanner (Hamamatsu, Bridgewater, NJ). Scanned images were viewed using the Hamamatsu NDP.view2 software to score antibody staining.
